# Regulation of Endoplasmic Reticulum-Associated Protein Degradation (ERAD) by Ubiquitin

**DOI:** 10.3390/cells3030824

**Published:** 2014-08-05

**Authors:** Leticia Lemus, Veit Goder

**Affiliations:** Department of Genetics, University of Seville, Av. Reina Mercedes 6, 41012 Seville, Spain

**Keywords:** ubiquitin, ERAD, Cdc48, p97, proteasome, E3 ubiquitin ligase, deubiquitinase

## Abstract

Quality control of protein folding inside the endoplasmic reticulum (ER) includes chaperone-mediated assistance in folding and the selective targeting of terminally misfolded species to a pathway called ER-associated protein degradation, or simply ERAD. Once selected for ERAD, substrates will be transported (back) into the cytosol, a step called retrotranslocation. Although still ill defined, retrotranslocation likely involves a protein conducting channel that is in part formed by specific membrane-embedded E3 ubiquitin ligases. Early during retrotranslocation, reversible self-ubiquitination of these ligases is thought to aid in initiation of substrate transfer across the membrane. Once being at least partially exposed to the cytosol, substrates will become ubiquitinated on the cytosolic side of the ER membrane by the same E3 ubiquitin ligases. Ubiquitin on substrates was originally thought to be a permanent modification that (1) promotes late steps of retrotranslocation by recruiting the energy-providing ATPase Cdc48p/p97 via binding to its associated adaptor proteins and that (2) serves to target substrates to the proteasome. Recently it became evident, however, that the poly-ubiquitin chains (PUCs) on ERAD substrates are often subject to extensive remodeling, or processing, at several stages during ERAD. This review recapitulates the current knowledge and recent findings about PUC processing on ERAD substrates and ubiquitination of ERAD machinery components and discusses their functional consequences.

## 1. ER Protein Quality Control and ERAD

Up to 30% of all proteins in the eukaryotic cell are targeted to the secretory pathway. The initial translocation across or insertion into the membrane of the endoplasmic reticulum (ER) is mediated by the protein conducting Sec61-channel or “translocon”. It has only a narrow pore and requires that proteins cross the membrane in an unfolded state [1]. Consequently, proteins fold post-translocationally inside the ER, a process that is aided and controlled by a battery of chaperones. Folding, however, is still inefficient and many proteins fail to acquire their native three-dimensional structure. A major cellular pathway is dedicated to remove terminally misfolded proteins from the lumen or the membrane of the ER and targets them for regulated degradation. The pathway is conserved among eukaryotes and has been named ER-associated (protein) degradation, or simply ERAD [2,3,4].

During ERAD, misfolded proteins are retrotranslocated back to the cytosol where they will undergo ubiquitination. Initially it was thought that ubiquitination of ERAD substrates serves exclusively as a tag for ultimate recognition by the proteasome. However, as it will be discussed in this review, accumulating evidence shows that ubiquination has various additional roles for ERAD. This is mainly linked to the findings that deubiquitinating enzymes (DUBs) are required for ERAD. DUBs remove single or multiple ubiquitins from polyubiquitin chains (PUCs), implying that PUCs on substrates are processed prior to their removal upon protein degradation by the proteasome. Moreover, it appears that PUCs can be processed during ERAD at several stages. This includes initial phases of ERAD where proteins are triaged for degradation and subsequent phases such as protein retrotranslocation across the ER membrane. Finally, ubiquitin also appears to be important for ERAD by regulating the composition or conformation of machinery components thereby controlling ERAD efficiency and substrate retrotranslocation.

## 2. Ubiquitination in ERAD

Ubiquitination is a post-translational modification that occurs in the cytosol and nucleoplasm of eukaryotic cells and requires a cascade of reactions [5,6,7]. Briefly, ubiquitin is activated by its ATP-dependent C-terminal coupling to the thiol group of a specific cysteine of a so-called E1 enzyme. Subsequently, ubiquitin is transferred to a specific cysteine of an E2 enzyme and finally to the substrate, usually to the ε-amino group of internal lysines, which is presented to the E2-linked ubiquitin by an E3 ubiquitin ligase. In yeast, there are one E1 enzyme, thirteen E2 enzymes and a much larger number of E3 ligases. A similar ratio between these families is found in mammalian cells although more individual enzymes from each class are present. Whereas E1 and E2 enzymes are common to many individual reactions, the large number of E3 ligases is likely because many of them are specific for only one or few particular cellular substrate(s). In contrast, only a small number of E3 ligases have a function in ERAD but they catalyze the ubiquitination of a large variety of distinct substrates. Consequently, these E3 ligases have much broader specificities for substrates.

In yeast, the known E3 ligases with a function in ERAD are Doa10p and Hrd1p. They are involved in the degradation of essentially all identified ERAD substrates so far and they are the core components of two individual protein complexes consisting of several membrane integrated proteins and associated components in the ER lumen as well in the cytosol. These complexes have been named accordingly, Doa10-complex and Hrd1-complex [8,9,10]. Doa10p and Hrd1p themselves are multispanning ER membrane proteins and belong to the family of RING ligases, named after the presence of the RING (Really Interesting New Gene) domain on their cytosolic C-terminus which catalyzes the transfer of ubiquitin from the E2 directly to the substrate [7]. The E2 enzymes with a function in ERAD are Ubc6p and Ubc7p (for Doa10p) and Ubc1p and Ubc7p (for Hrd1p) [11]. Mammalian cells possess ERAD-specific membrane protein complexes with homologues components and almost identical functions compared to yeast but also have additional E3 ligases with roles in ERAD that are less well characterized, reviewed in [11]. Interestingly, many of the key components that mediate ERAD not only have a role in clearing the ER from misfolded proteins but they are also involved in regulating metabolic pathways. For instance, the production of sterols in eukaryotic cells is tightly controlled at several points by metabolite-triggered ERAD of regulatory elements [8,12]. These observations illustrate that a common pathway can serve distinct functional purposes.

**Figure 1:**
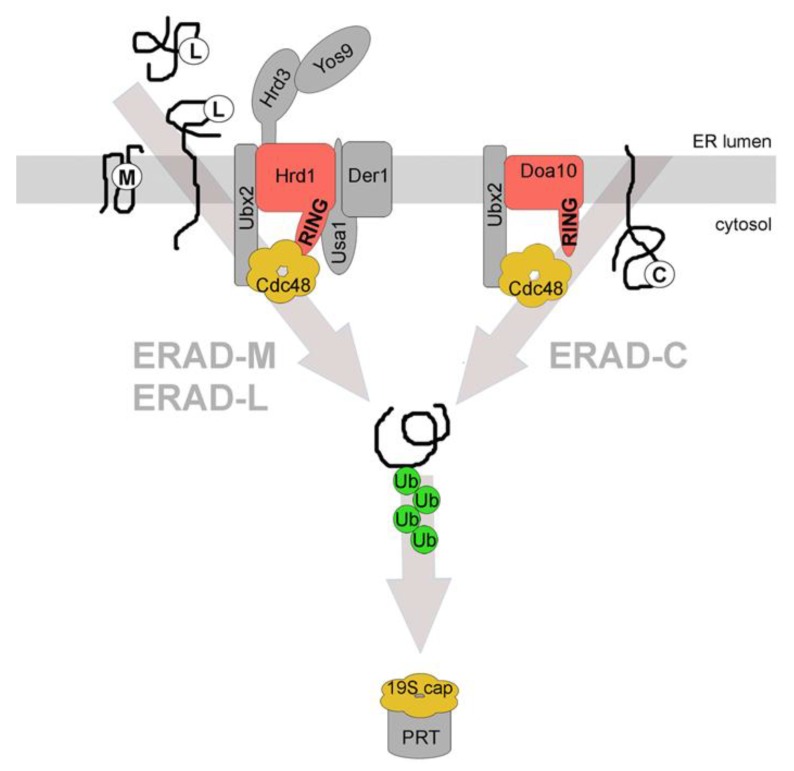
Distinct endoplasmic reticulum-associated degradation (ERAD) pathways. Three main ERAD pathways in yeast are classified based on substrates and the components that are involved in their degradation. ERAD-L degrades membrane integrated or soluble proteins with misfolded domains in the ER lumen, marked with (L). All depicted constituents of the Hrd1-complex are required for the efficient degradation of these substrates. ERAD-M degrades membrane integrated proteins with misfolded regions in their transmembrane domain(s), marked with (M). Proteins of this class are degraded via the Hrd1-complex but do not require Usa1p and Der1p for efficient degradation. ERAD-C degrades membrane integrated proteins with misfolded domains in the cytoplasm, marked with (C). These proteins are degraded via the Doa10-complex. All ERAD pathways require cytosolic Cdc48p for substrate retrotranslocation and extraction from the ER membrane. The substrate is ubiquitinated by the E3 ligases during or after retrotranslocation and is targeted to the proteasome (PRT) for degradation. Cdc48p and the 19S cap of the proteasome have structural and functional similarities. The classification for the different ERAD pathways also exists in mammalian cells albeit it is less stringent. RING = RING domain of the E3 ligases that are shown in red. The associated constituents of the individual complexes are shown in grey. Ub = ubiquitin. See text for details.

It has been observed that the location of misfolded domain(s) on ERAD substrates is decisive for which ERAD machinery complex is used for their subsequent degradation. Based on this, three major ERAD pathways have been defined (Figure 1). Membrane proteins with a misfolded cytosolic domain are generally targeted to the Doa10-complex, a pathway termed ERAD-C (C for cytosolic) [13]. Membrane proteins with a misfolded luminal domain are generally targeted to the Hrd1-complex, a pathway termed ERAD-L (L for luminal) [13]. The same pathway degrades soluble misfolded proteins of the ER lumen. Finally, membrane proteins with a misfolded region in their transmembrane domain(s) are also targeted to the Hrd1-complex. However, in this case, fewer associated components are required for the degradation of these substrates; this pathway was termed ERAD-M (M for membrane) [14,15,16]. The correlation between substrate class and “choice” of pathway is strongest in yeast but is also found in mammalian cells, albeit to a lesser extent.

A common mechanism of how individual misfolded proteins in the ER are first recognized is not known but it likely involves several criteria such as the exposure of hydrophobic patches and the dwell time of binding to chaperones. The selected substrates are then targeted to the membrane-embedded E3 ligase complexes where they undergo ubiquitination on their cytosolically exposed protein domains during or after retrotranslocation.

Generally, the 76 amino acid long ubiquitin is coupled to mostly ε-amino groups of internal lysine residues in substrates, via an isopeptide bond. In particularly prepared model substrates for ERAD that lacked lysine residues, ubiquitin could also be coupled to the hydroxyl groups of serine or threonine side chains, or to the sulfhydryl group of cysteine, forming ester bonds [17,18,19]. After initial (mono-)ubiquitination, substrates can be further modified with additional ubiquitins leading to the formation of PUCs. PUCs can occur in a very large number of different chain conformations because ubiquitins can attach to one another in several ways. The C-terminal glycine of the donor ubiquitin can be attached to the α-amino group of the N-terminus of the acceptor ubiquitin, *via* a peptide bond, or to any of its seven internal lysines (K6, K11, K27, K29, K33, K48 and K63), via an isopeptide bond. A PUC with identical linkages between the ubiquitin moieties is called homotypic whereas different linkages lead to the formation of heterotypic chains, reviewed in [20].

It is currently unclear whether the ERAD-specific E3 ligases mono-ubiquitinate substrates or whether they generate PUCs with specific linkage types or chain lengths. *In vitro* experiments with components from yeast and mammalian cells suggested that poly-ubiquitin chains can form on substrates in a single step. For instance, ERAD E3 ligases could catalyze the *en bloc* transfer of preassembled PUCs from E2 enzymes directly to substrates [21,22]. E2-specific cofactors are also thought to stimulate PUC formation by increasing the E2 activity [23]. Finally, certain E2 cofactors, such as Cue1p in yeast, have been shown to stabilize PUCs by binding to them and are therefore thought to promote PUC formation on substrates [24].

## 3. Deubiquitination in ERAD

Deubiquitinating enzymes (DUBs) comprise a large protein family that can be subdivided further into five subfamilies: ubiquitin C-terminal hydrolases (UCHs), ubiquitin-specific proteases (USPs), ovarian tumor proteases (OTUs), Josephins (Josephins) and JAB1/MPN/MOV34 (JAMMs) [25]. Only a few identified DUBs have so far been shown to be clearly relevant for ERAD. They are, however, not restricted to a particular family as they belong to the USPs (Usp13, Usp25), Josephins (Ataxin3), and OTUs (YOD1) families [26,27,28,29,30]. Additional DUBs might play a yet insufficiently characterized role during ERAD, such as USP19, USP50 and VCPIP1 [31,32,33].

Although a detailed understanding for the function of DUBs in ERAD is far from being complete, the general emerging picture is that their actions allow the constant remodeling of PUCs on substrates in combination with ubiquitination reactions. Such PUC remodeling would allow more selective and efficient recognition of substrates and the regulation of transport to the proteasome for their subsequent turnover. Recent evidence indicates that similar cycles of ubiquitination and deubiquitination reactions also occur on ERAD machinery components. This might be yet another important mechanism for the regulation of ERAD. In the following, examples from recent studies with specific relevance to these mechanisms will be discussed in more detail.

## 4. PUC Processing as a Mechanism for ER Membrane Protein Quality Control

**Figure 2:**
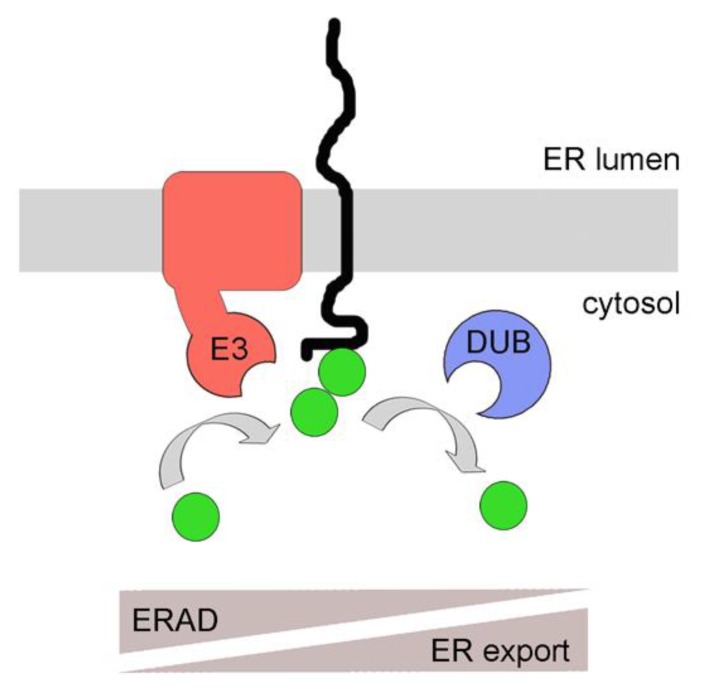
A model for poly-ubiquitin chain (PUC) processing as a mechanism for coupling ER protein quality control with ERAD. Membrane proteins with exposed cytosolic domains are ubiquitinated by E3 ligases. Due to topological confinement of membrane proteins and membrane integrated E3 ligases to the same bilayer, ubiquitination might occur frequently, even on correctly folded proteins. DUBs can remove ubiquitins, favoring ER export of correctly folded species. Reoccurring ubiquitination due to prolonged ER retention as a result of misfolding (in the cytosol or ER lumen) will favor the assembly of PUCs to induce targeting to the proteasome, thereby favoring ERAD. A similar mechanism might occur for soluble proteins prior to their post-translational translocation across the ER membrane, a pathway called prERAD. See text for details. E3 = E3 ligase. DUB = deubiquitinase. Filled green circles: ubiquitin.

ER membrane proteins are often directly accessible to the cytosolic ubiquitination machinery through their cytosolic domains and ubiquitination of these domains could result in a direct signal for ERAD. Recently, however, ubiquitination of cytosolic domains of ER membrane proteins was proposed to have a function prior to their degradation and would instead be involved in their quality control [34]. In this particular case, the authors investigated the traffic of mammalian lipoprotein receptor-related protein-6 (LRP6) [35]. LRP6 is a type I membrane protein with a large luminal domain and a shorter cytoplasmic tail. It is constitutively palmitoylated at one or possibly two juxtamembranous cysteines, and is normally targeted to the plasma membrane for its function in Wnt signaling. Abrogation of palmitoylation by mutating the critical cysteine residues resulted in mono-ubiquitination on a proximal lysine residue [35]. However, mono-ubiquitination did not trigger ERAD but resulted in protein retention inside the ER. The authors suggested that the observed initial mono-ubiquitination would be a first and critical step in the quality control of particular membrane proteins and would prevent the protein from being prematurely exported from the ER. In addition to that, mono-ubiquitinated substrate would also allow recruitment of yet-to-be defined cytosolic quality control components, potentially including chaperones and folding sensors [34]. Prolonged retention of the protein in the ER will lead to the extension of mono-ubiquitin by E3 ligases and to the formation of PUCs. Initially this could be reversed by DUBs but eventually, in case that protein folding attempts repeatedly fail, PUCs would form which are recognized by the ERAD system (Figure 2). Clearly, the model is still very hypothetical and needs more experimental backup for validation. For instance, it is still unclear which E3 ligases or DUBs are involved in modifying LRP6. Despite all these uncertainties, the model is appealing with the concept that E3 ligases and DUBs are involved in modifying PUCs on cytosolic domains of ER membrane proteins in a similar manner and with similar consequences like ER luminal UDP-glucose:glycoprotein glucosyltransferase (GT) and Glucosidase II, which are involved in modifying N-glycans on proteins for quality control in the ER lumen [36].

Another recent study highlighted the kinetic aspect of protein quality control *versus* ERAD and the roles of E3 ligases and DUBs in these processes [37]. The authors addressed an issue that is particular relevant for E3 ligases involved in ERAD since they are very promiscuous due to the large number of substrates they have to deal with. It is predictable that these ligases posses different affinities to particular substrates, yet all substrates have to be efficiently ubiquitinated for degradation. Whereas soluble ER luminal proteins are only getting access to the E3 ligases after being selected for ERAD and targeted to the membrane, ER membrane proteins have to fold in the neighborhood of these E3 ligases. Thus, the chances for encounter and ubiquitination are high even if the substrate might not end up terminally misfolded. Due to the limitations to purify defined misfolded proteins the authors used a viral system where correctly folded mammalian CD4, a membrane protein with a sizable cytosolic tail, is targeted by the HIV-encoded membrane protein Vpu to the cytosolic SCF E3 ligase, leading to ubiquitination, extraction of CD4 from the ER membrane and proteasomal degradation [37]. This system recapitulates cellular ERAD. It turned out that the observed ubiquitination state of particular CD4 mutants could only be explained by kinetic modeling incorporating DUB activity. This was supported by subsequent *in vitro* and *in vivo* experiments using DUB inhibitors [37]. Based on their observations the authors proposed a model where frequent ubiquitination is occurring on many cellular substrates, in particular on those that are restricted to the membrane and thus are in constant vicinity to membrane-embedded E3 ligases, such as those involved in ERAD. Properly folded substrates would have weaker affinity to E3 ligases resulting in shorter PUCs. Importantly, these PUCs would be further shortened or removed by DUBs, preventing protein degradation. In contrast, substrates with higher dwell time with an E3 ligase would result in longer PUCs, ultimately resulting in proteasomal degradation of the substrate (Figure 2). This system would thus resemble important aspects of the earlier discussed quality control mechanism proposed for mammalian LRP6 [34].

A recent report revealed yet another mechanism for the connection of ER quality control and ERAD [38]. The authors proposed that ER proteins are quality controlled even prior to membrane insertion or translocation, a mechanism they called prERAD [38]. This mechanism seems to occur when proteins translocate post-translationally, *i.e.*, after protein synthesis. In such cases, some proteins associate transiently with the cytosolic leaflet of the ER membrane. They are then scrutinized by the E3 ligase Doa10p and, interestingly, by the DUB Ubp1p [38]. Again, it was proposed that an interplay between ubiquitination and deubiquitination reactions as a result of yet unknown determinants of protein quality would determine whether the protein will be degraded by the proteasome or translocated across or inserted into the ER membrane [38].

## 5. PUC Processing as a Mechanism to Promote Protein Retrotranslocation

**Figure 3:**
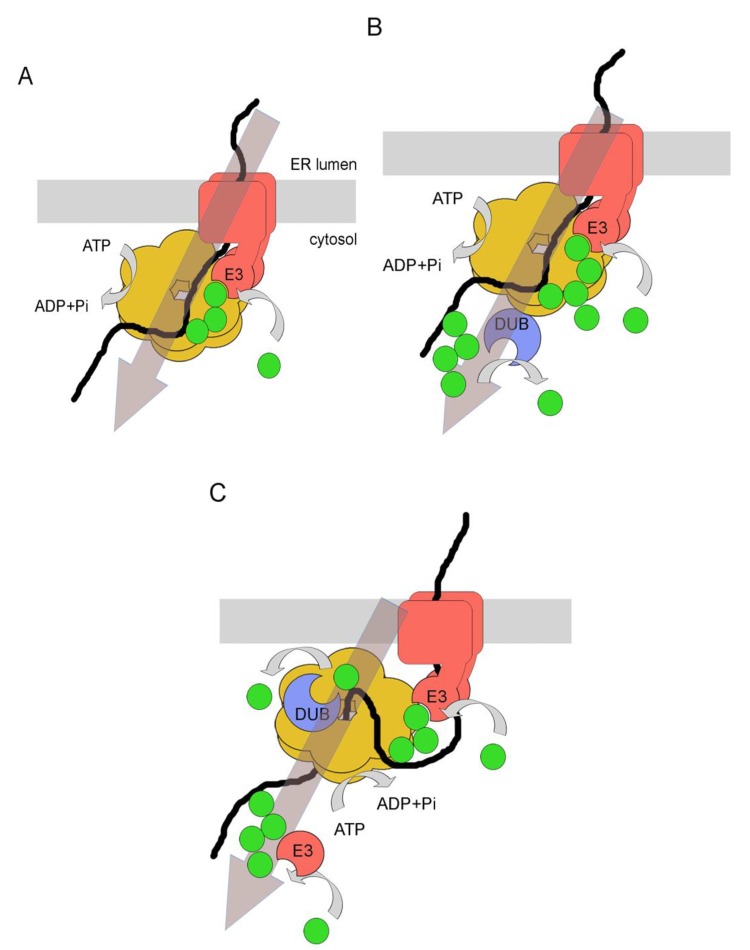
Models for PUC processing as a mechanism for protein retrotranslocation. (**A**) Ubiquitination of the substrate on the cytosolic side of the ER membrane by an E3 ligase promotes binding to the hexameric Cdc48p, via its ubiquitin-binding cofactors Npl4p and Ufd1p. ATP hydrolysis by Cdc48p is thought to promote the complete retrotranslocation and substrate extraction from the ER membrane. See text for details. (**B**) In the mammalian system, the p97-associated DUB Ataxin-3 acts downstream of protein retrotranslocation and might promote the removal of ubiquitins from longer PUCs. This is thought to be required for the generation of PUCs with optimal binding affinities to proteasomal shuttle factors or to the proteasome itself. See text for details. (**C**) The p97-associated DUB YOD1 is thought to act upstream of protein retrotranslocation. Its activity was suggested to be needed for the complete removal of PUCs to allow passage of the substrate through the pore of p97. ATP hydrolysis would then couple protein retrotranslocation with protein unfolding. Subsequently, reubiquitination by an E3 ligase would allow substrate targeting to the proteasome. See text for details.

Shortly after the discovery of ERAD it became clear that ubiquitination of substrates has an important role not only for their ultimate degradation by also for their retrotranslocation [39,40,41]. Major insights into the mechanism that links ubiquitination with retrotanslocation came from the discovery that Cdc48p (p97 in mammals), a well-conserved hexameric AAA-ATPase, is involved in ERAD [42,43]. Cdc48p is a very abundant protein found in the cytosol and nucleus, and it has multiple cellular functions beyond ERAD. They involve control of the cell cycle, membrane fusion, autophagy and DNA repair, reviewed in [44]. The distinct cellular roles of Cdc48p are determined through binding to different co-factors. This is particularly relevant for the recruitment of Cdc48p to ubiquitin. Although it has been shown that Cdc48p itself can bind to ubiquitin via its N-domain, its ERAD-specific co-factors Npl4p and Ufd1p (Npl4 and Ufd1) provide additional binding sites to ubiquitin [45]. For instance, mammalian Npl4 possesses a ubiquitin-binding zinc finger motif (which is not present in the yeast orthologue) whereas Ufd1p has a conserved so-called UT3 motif. This UT3 motif makes Ufd1p to prefer the binding to poly-ubiquitin over mono-ubiquitin, suggesting that some PUCs have formed on substrates at the stage when it connects to Cdc48p [46]. Important for the proposed role of Cdc48p in substrate retrotranslocation is also the finding that it is recruited to the ER membrane via direct binding to E3 ligases such as Hrd1p (and gp78) or, independently, via binding to one of its co-factors and integral membrane protein Ubx2p (UbxD8), which is part of both the Hrd1- and Doa10-complexes [47,48,49,50]. Thus, by binding to ubiquinated substrates on the one hand and to the ER membrane on the other hand, Cdc48p might pull substrates out of or across the membrane like a molecular ratchet, fueled by repeating cycles of ATP hydrolysis and the accompanying conformational changes (Figure 3A) [51,52]. Whether substrate ubiquitination generally precedes binding to Cdc48p and its co-factors or whether initial binding of non-ubiquitinated substrates to Cdc48p would facilitate their subsequent ubiquitination by E3 ligases, especially with respect to the build-up of PUCs on substrates, is still not clear. However, several recent reports place Cdc48p in the center of the processing of PUCs. This has important implications for mechanistic aspects of ERAD and provides new models for how substrates are retrotranslocated and how they are targeted to the proteasome after retrotranslocation.

First studies that connected DUB activity to Cdc48p/p97 were not related to ERAD [53,54,55]. However, subsequent reports from mammalian cells showed that specific DUBs do have a role in ERAD [26,27,28,56]. Somewhat counterintuitive at first glance, DUB activity was found to be a positive regulator for substrate degradation [26,28]. The first p97-associated DUB that was proposed to have a function in ERAD was Ataxin-3 [26]. The authors of this study proposed that the DUB would act downstream of p97-mediated retrotranslocation and would trim the PUCs on substrates if necessary to maintain an optimal signal for targeting to the proteasome, thereby streamlining protein retrotranslocation with their recognition by the proteasome (Figure 3B) [57]. Additional data came from work with YOD1, another DUB that was found to be associated with p97 [28]. YOD1 belongs to the OTU family of DUBs and was shown to prevent substrate degradation when expressed as a dominant negative version (YOD1 C160S) [28]. Important for mechanistic aspects of retrotranslocation, the model substrate used in this study was found poly-ubiquitinated and still associated with the ER membrane when the mutant DUB was expressed, suggesting that DUB activity was required to allow p97-mediated completion of substrate retrotranslocation [28]. To address this model in more detail, the same group expressed a highly active viral DUB from the Epstein-Barr virus (EBV-DUB) in the same system [58]. Two crucial observations were made. First, the degradation of various ERAD substrates was blocked under these conditions. Second, one particular substrate accumulated as a deglycosylated form in the cytosol, showing that is has been retrotranslocated but has not been forwarded to the proteasome [58]. The authors concluded that deubiquitination would influence ERAD, and in particular retrotranslocation, at distinct cellular stages, upstream and downstream of p97. In one model, initial ubiquitination of substrates by the ERAD ubiquitination machinery would trigger direct binding to p97. DUB-mediated PUC trimming or removal would then be needed to allow p97-mediated retrotranslocation. Subsequently, reubiquitination of substrates would facilitate their transfer to downstream components and targeting to the proteasome (Figure 3C) [59].

One speculative part of that model suggests that DUB activity upstream of p97 is required for threading the substrate through the pore in the center of p97 [28,58]. The evidence for this model is scarce at the moment and there are arguments in its favor and against it. The arguments in its favor concern the functional and structural analogies between Cdc48p/p97 and related AAA-ATPases like the regulatory particle of the proteasome and the *E.coli* protein ClpB. For instance, the regulatory particle of the proteasome threads substrates through its interior pore in an ATP-dependent manner and feeds it directly into the physically associated proteolytic chamber, the core particle, thereby coupling unfolding of the substrates with its subsequent degradation [60]. A similar mechanism of substrate translocation through the channel was shown for ClpB although, in contrast to its homologs ClpA and ClpX, it does not associate with the bacterial proteolytic chamber ClpP [61]. Thus, Cdc48p/p97-mediated protein unfolding might resemble a situation found in ClpB. Arguments against the threading model include structural studies with p97 mutants [62]. Only the central pore residues contributed by the D2 domain of the six p97 monomers but not the D1 domain were found to affect ERAD. To account for this phenomenon, it was proposed that substrates might enter and exit trough the same side of the channel, namely the part that is formed by the assembly of the D2 domains [62]. Alternatively, substrates might enter through the D2 pore and exit laterally between the D1 and D2 domains but proof for either of these models is currently lacking [62]. The threading model has also to be looked at with caution to account for the fact that some ERAD model substrates can apparently be retrotranslocated across the ER membrane in a partially or completely folded state, too big to envision that they would pass through the p97 pore during that process [63,64]. Although it has not been directly addressed whether p97 is involved in the retrotranslocation of these folded model substrates, it is required for ERAD of directly related proteins [65]. Therefore, threading of substrates through the pore of Cdc48p/p97 might not always be mandatory. Clearly, more research is needed to clarify the mechanistic aspects of how Cdc48p/p97 promotes the retrotranlocation of substrates.

## 6. PUC Processing as a Mechanism for Targeting of Substrates to the Proteasome

Considering the currently available data for PUC processing on ERAD substrates during retrotranslocation it appears not unlikely that those PUCs that serve for recognition by the proteasome are generated specifically at later stages during ERAD, potentially after partial or complete removal of initial PUCs. The precise order of events might also depend on the specific cellular substrate and its requirements for retrotranslocation. PUCs for the recognition by the proteasome could be generated by the same ERAD-specific E3-ligases and DUBs involved in the initial retrotranslocation, or by additional components. Due to the general scarcity of available data for how PUCs with specific lengths and linkages are generated, these assumptions remain entirely speculative.

However, some models for how PUC processing at later stages during ERAD could ensure efficient substrate targeting to the proteasome have been put forward. They are mainly based on data obtained from work with yeast and, more specifically, from the functional characterization of Cdc48p-associated co-factors and of cytosolic “shuttle” factors of ERAD substrates. Important insight came from the discovery that Cdc48p recruits Ufd2p (Ufd2a), a specialized E3 ligase with an unusual ligase domain, the U-box, instead of a RING domain [66]. In connection with E1, E2 and E3 enzymes, Ufd2p promotes the extension of short ubiquitin chains, an activity that coined the term “E4 enzyme” [66]. Association of Cdc48p to Ufd2p leads to an increase in the binding affinity of Ufd2p for short PUCs, stimulates its E4 activity but also prevents excessive chain extension [67]. Importantly, the extended Ufd2p-generated PUCs contain preferentially K48-linkages [66,68]. Ufd2p was also reported to be required for the ERAD of several tested model substrates [67,69]. Ufd2p could thus mediate the generation of PUCs on ERAD substrates with an optimal length and linkage for proteasomal degradation.

An appealing mechanism of how Ufd2p would couple PUC extension on substrates with their efficient targeting to the proteasome was proposed by combining these findings with earlier data that revealed the involvement of specific cytosolic factors such as Rad23p and Dsk2p in ERAD [70,71]. The cytosolic factors Rad23p and Dsk2p were identified by a genetic screen in yeast for novel components with a function in ERAD [72]. A common structural hallmark is that they contain one or more of both an UBL (ubiquitin-like) and an UBA (ubiquitin-associated) domain. These features are conserved and were providing the name “UBL-UBA” to three protein families named after Rad23, Dsk2 and Ddi1, reviewed in [60]. So far, only members of the Rad23 and Dsk2 protein families were shown to play roles in ERAD. The UBL domain of Rad23p (HHR23A and HHR23B) was found to bind to Ufd2p or to the proteasomal ubiquitin receptor Rpn10p (hRpn10/S5a) in an exclusive manner [73,74,75]. In addition, the UBA domains of HHR23A and Rad23p were found to bind preferentially to PUCs with a chain length of 4-6 ubiquitins [76]. Binding to UBA domains was proposed to be an additional mechanism to regulate PUC length and to prevent excessive chain extension by E3 ligases [77,78]. Dual binding to the proteasome or to Cdc48p–associated components with their UBL domain and to substrates with their UBA domain is thought to put UBL-UBA proteins in the position to link specific PUCs on substrates with their targeting to the proteasome [67]. Although this simple model is not entirely free of controversy [79], there is also strong experimental evidence supporting it [80].

**Figure 4:**
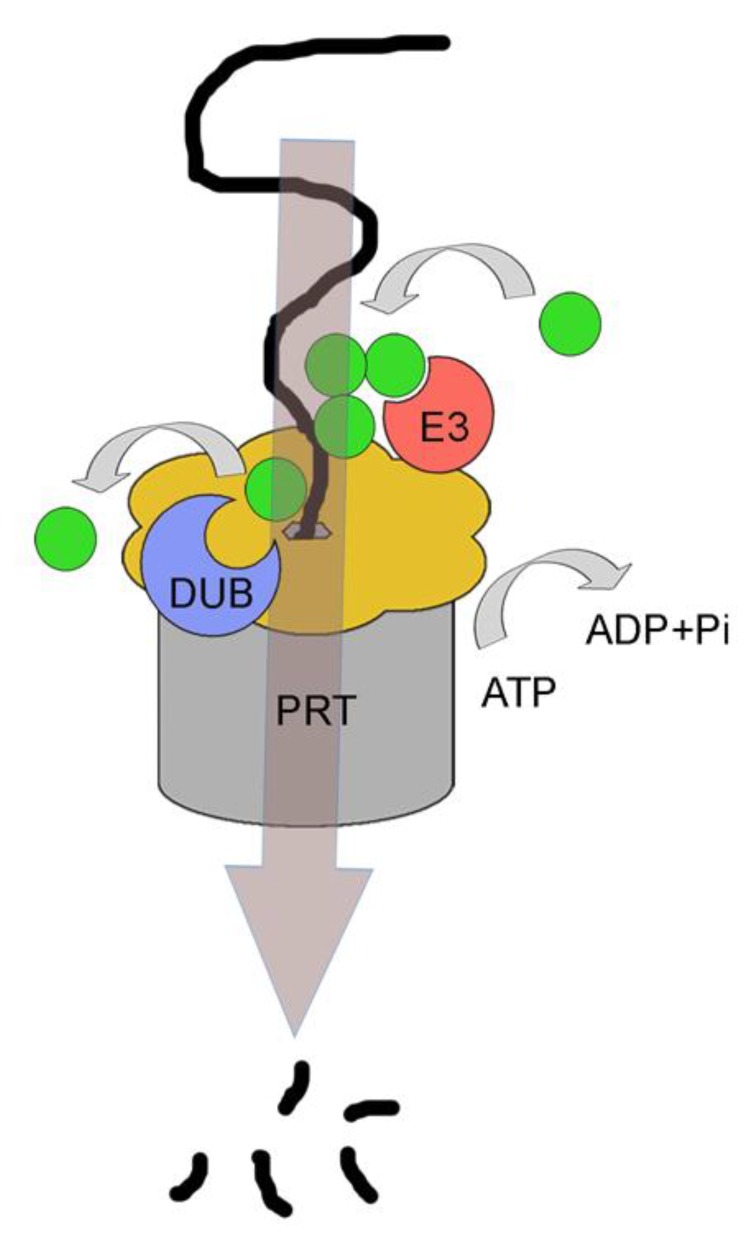
The 19S cap of the proteasome has functional and structural similarities with Cdc48p/p97. Like Cdc48p/p97, the hexameric 19S cap of the proteasome binds simultaneously to E3 ligases and to DUBs, suggesting that PUCs are processed at this stage, immediately before substrate degradation. This could be needed for the transfer of the substrate from the periphery of the proteasomal lid through its central pore and further into the proteolytic chamber of the proteasome. Immediately before passage through the pore of the l9S cap, structural data suggest that all PUCs are to be removed. See text for details.

Interestingly, the regulatory particle of the proteasome is connected not only to DUBs but also to E3-ligases (Figure 4). Together they might thus have similar roles in PUC processing like the Cdc48p/p97-associated ubiquitin-modifying enzymes [70]. From the E3 ligases with reported associations with the proteasome, Hul5p (Hul5) is the most prominent example with stochiometric amounts bound to the proteasome in both yeast and mammals [81]. Other proteasome-associated E3 ligases include Ubr1p, Ufd4p and SCF, for review see [82]. Relevant for ERAD, Hul5p was shown to be required for the complete degradation of membrane-bound ERAD substrates [83]. Hul5p was shown to be tightly associated with the DUB Ubp6 (USP14) on the proteasome and both enzymes are thought to regulate the efficient degradation of substrates by keeping the balance of chain-extending and chain-trimming activities [81].

Complete removal of PUCs at the proteasome which would not be coupled to the insertion of substrates into the proteolytic chamber was also thought to provide an “ultimate rescue” of aberrantly ubiquitinated substrates [84]. Such a mechanism would thus be similar to the earlier described quality control of ER membrane proteins where aberrantly ubiquitinated proteins can be spared from degradation by the activity of DUBs. However, although such a “rescue” might be useful for cytosolic or nuclear proteins, or for ER membrane proteins as part of their quality control, it should be of no use for ERAD substrates after they have been completely retrotranslocated or extracted from the membrane, because they could not re-enter the ER. Translocation of proteins into the ER lumen depends on an N-terminal signal sequence which is cleaved during or after translocation [1]. This impedes “a second” translocation into the ER. Membrane proteins, especially those with several transmembrane domains, are normally inserted into the ER membrane co-translationally, *i.e.*, with the synthesizing ribosome being docked onto the translocon [85]. Once extracted, “a second” post-translational insertion process seems mechanistically and energetically highly unlikely. Thus, specific combinations of DUBs and E3 ligases on the proteasome might allow for a quality control mechanism for certain substrates only. In general, however, PUC processing at the proteasome might be largely needed for initial substrate binding and for subsequent movement of substrates into the proteolytic chamber. For the first step, the generation of optimal PUCs was proposed to efficiently reduce the dissociation rates from ubiquitin receptors of the proteasome regulatory particle, Rpn10p (PSMD4) and Rpn13p (ADRM1) [81]. Rpn13p was shown to bind preferentially K48-linked-diubiquitins in comparison to other types of linkages [86,87]. Alternatively, PUC processing at this stage might also contribute to non-proteolytic functions of the proteasome [88,89].

Still, there is too little knowledge about the effects of specific PUCs with regard to the proteasomal degradation of a substrate. PUCs with homotypic K48-linked chains with at least four ubiquitins are known to provide an efficient signal for recognition by the proteasomal ubiquitin receptors [90]. However, as is becomes more apparent that the potential variety of PUCs with distinctive lengths and linkages has likely functional relevance *in vivo*, it is already evident that PUCs that encode a signal for degradation by the proteasome come in more flavors than 4–6 ubiquitins with a homotypic K48-linkage [91,92]. With the exception of K63-linkages, practically all possible modes of conjugations of ubiquitins in PUCs were found to support proteasomal degradation *in vivo* [93]. In selected cases, single ubiquitins on substrates were also shown to be sufficient for proteasomal degradation [94]. In addition, recent data show that branched PUCs with K11- and K48-linked chains provide a better signal for degradation by the proteasome than homotypic K11- or K48-linked chains [95]. Thus, a diversity of distinct PUCs can catalyze the degradation of substrates and the precise length and linkage of a particular PUC might influence kinetic aspects of turnover of particular substrates.

Apart from being the ultimate degradation machinery for ERAD substrates, the proteasome itself might actively participate in protein retrotranslocation or extraction of proteins from the ER membrane, similar to Cdc48p/p97. This is not too surprising, considering the remarkable structural and functional similarities between the regulatory particle of the proteasome and Cdc48p/p97. Both protein complexes can associate with multiple co-factors that can bind and process PUCs and both utilize ATP hydrolysis to undergo large conformational changes for the unfolding/movement of substrates, reviewed in [96]. The precise role of the proteasome in retrotranslocation likely depends on the substrate. The tail-anchored membrane protein Ubc6p, an E2 protein for the E3 ligase Doa10p, is continuously degraded by ERAD and its extraction from the ER membrane directly depends on the proteasome [97,98]. Thus, a recruitment of the proteasome to the ER membrane could be catalyzed by direct interaction with the cytosolic and ubiquitinated part of the ERAD substrates.

Interestingly, the proteasome was also found to bind to the ER membrane via association with the translocon [99,100]. The translocon, which constitutes the protein import machinery for the ER, was long considered a prime suspect for being the retrotranslocation channel as well [101,102,103,104]. Thus, there was a physiological relevance for the association with the proteasome. However, recent structural data suggest that the Sec61-channel cannot be easily accessed from the ER lumen for protein retrotranslocation [105]. Instead, members of the ER membrane-embedded ERAD-specific ubiquitination machineries such as Hrd1p and Der1p in yeast, and homologs in mammals, are more likely to constitute the conduit for protein retrotranslocation [106,107,108,109]. Interestingly, a recent report documents a role for the Hrd1-complex in freeing potentially obstructed translocons from stalled nascent chains by directing them to ERAD [110]. It could thus be that the observed association of the proteasome with the translocon is an indirect consequence of the ubiquitination of channel obstructing substrates by ERAD E3 ligases. In support of such a scenario, huge protein complexes, encompassing ERAD ubiquitination machinery components, ER luminal chaperones, cytosolic Cdc48p and the proteasome were shown to assemble under specific circumstances [111,112].

## 7. Ubiquitination and Deubiquitination of ERAD Machinery Components

**Figure 5:**
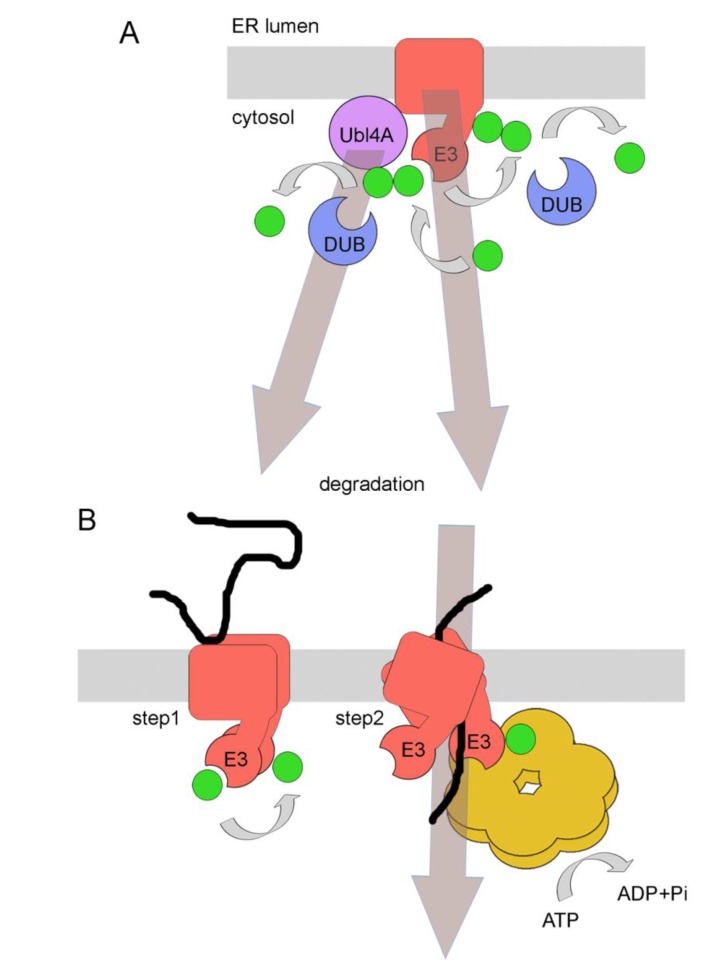
Models for ubiquitination and deubiquitination of ERAD machinery components as a mechanism to regulate ERAD. (**A**) Regulation through degradation. In the mammalian system, ubiquitination of the ERAD component Ubl4A by the E3 ligase gp78 leads to the degradation of the associated shuttling chaperone Bag6, thereby reducing efficient substrate targeting to the proteasome. Ubl4A ubiquitination is reversed by the DUB Usp13. A similar mechanism involves the self-ubiquitination of Hrd1p in yeast in absence of Hrd3p and Usa1p, leading to effective degradation of the E3 ligase. No DUB is currently known in this system. See text for details. (**B**) Regulation through conformational changes. Binding of a substrate to Hrd1p in the ER lumen induces oligomerization and self-ubiquitination of the E3 ligase (step 1). Subsequently, ATP hydrolysis by bound Cdc48p would result in conformational changes in the ATPase, which will consequently result in conformational changes in Hrd1p oligomers. This, in turn, would push the substrate through a postulated channel formed by Hrd1p oligomers (together with associated components like Der1p) until it is partially exposed to the cytosol (step 2). See text for details.

Several recent studies started to reveal that ubiquitination and deubiquitination also occurs on ERAD machinery components [29,107,113]. Self-ubiquitination and subsequent degradation as one mode of regulation of cellular ubiquitination machineries has been known for some time, reviewed in [114]. One such example relevant for ERAD is the regulation of the E3 ubiquitin ligase Hrd1p in yeast. Hrd1p undergoes self-ubiquitination and degradation in dependence on its binding partners Hrd3p (Sel1) and Usa1p (Usa1) [115,116,117] (Figure 5A). Such a scenario might also involve additional regulating elements like DUBs. In support of this, the DUB YOD1 was recently shown to affect the turnover of a non-ubiquitinated ERAD substrate in mammalian cells, implying that this DUB might act on ERAD machinery components [113]. Since YOD1 is also known to act on PUCs on substrates, these results suggest that certain DUBs can process ubiquitins or PUCs on substrates and on ERAD machinery components. Another example relevant for ERAD links the failure of DUB activity and the following degradation of an aberrantly ubiquitinated machinery component with downregulation of ERAD in mammalian cells [29]. The authors of the study investigated the regulation of the Bag6 chaperone system which keeps aggregation-prone retrotranslocated and membrane-extracted ERAD substrates in a soluble yet unfolded state for targeting to the proteasome [118]. Bag6 is bound to the ER membrane by multiple interactions, but binds specifically to gp78, a membrane-embedded E3 ligase similar to Hrd1p in yeast [119]. Strikingly, Usp13, a DUB associated to p97, affected the stability of the Bag6 complex. Failure of Usp13 activity lead to the polyubiquitantion of the Bag6 complex component Ubl4A by gp78 [29]. As a result, Bag6 is degraded, leading to downregulation of ERAD (Figure 5A).

In addition to these classical examples for the role of ubiquitin in targeting proteins for degradation, a distinct ubiquitination event of the machinery component Hrd1p was proposed to regulate ERAD in additional ways. Hrd1p likely forms the main component of the retrotranslocation channel by homo-oligomerization [107]. Interestingly, sustained substrate binding to Hrd1p on the luminal side of the ER membrane required its cytosolic E3 ligase activity [107]. The authors proposed a mechanism where substrate binding to Hrd1p on the luminal side leads to self-ubiquitination of Hrd1p on its cytosolic domain. Its ubiquitination, perhaps mono-ubiquitination, was proposed to activate the ATPase activity of the associated Cdc48p. Hydrolysis of ATP by Cdc48p would lead to conformational changes and structural rearrangements of the oligomerized Hrd1p, thereby aiding in initial phases of retrotranslocation until the substrate would be exposed to the cytosol and ubiquitinated by Hrd1p [107] (Figure 5B). Further work is needed to support this model, not the least because it remains currently unclear how deubiquitination of Hrd1p would be catalyzed.

Together, ubiquitination of ERAD machinery components can seemingly regulate ERAD in different ways. On the one hand, the regulation would be linked to cellular homeostasis (long term response) and involve the degradation of machinery components. On the other hand, regulation would be linked to mechanistic aspects of retrotranslocation (immediate response) and involve conformational changes of machinery components induced by activating ATP hydrolyzing machines like Cdc48p/p97.

## 8. Perspectives

The fact that DUBs can be positive regulators in ERAD has led to a new understanding for the roles of ubiquitin in the process of ERAD. It will be important to learn whether and at what stages of ERAD the processing of PUCs is following structural or kinetic aspects and what are the precise consequences with respect to the substrate. It seems clear that PUC processing on ERAD substrates most likely occurs when the substrates are connected to Cdc48p/p97 and to the proteasome during their journey to degradation. The temporal and spatial association of E3 ligases and DUBs with these huge ATP hydrolyzing machines is probably very dynamic and their activation is another parameter that will determine how PUCs are processed. It will be challenging to elucidate the molecular steps of PUC processing in a given environment and it would require the development of suitable *in vitro* assays with purified components. Similar systems should help to reveal the recently postulated functions of ubiquitination and deubiquitination in regulating ERAD machinery components through conformational changes. This could be very relevant for the mechanistic understanding of the actual retrotranslocation step in general but especially for soluble ER luminal proteins. These substrates do not expose any molecular “handle” to the cytosol prior to being at least partially retrotranslocated and the mechanisms that underlie the first steps of their transport back into the cytosol are therefore subject to intense research. The unambiguous identification of the exact composition of the postulated protein conducting channel would surely be a milestone in the field that would allow the tackling of these and related questions linked to a fascinating cellular pathway.
